# The Role of MYC2 Transcription Factors in Plant Secondary Metabolism and Stress Response Mechanisms

**DOI:** 10.3390/plants14081255

**Published:** 2025-04-20

**Authors:** Tuo Zeng, Han Su, Meiyang Wang, Jiefang He, Lei Gu, Hongcheng Wang, Xuye Du, Caiyun Wang, Bin Zhu

**Affiliations:** 1 Guizhou Key Laboratory of Forest Cultivation in Plateau Mountain, School of Life Sciences, Guizhou Normal University, Guiyang 550025, China; zengtuo@gznu.edu.cn (T.Z.); 221109040025@gznu.edu.cn (H.S.); jiefang.he@gznu.edu.cn (J.H.); leigu1216@nwafu.edu.cn (L.G.); wanghc@gznu.edu.cn (H.W.); duxuye@gznu.edu.cn (X.D.); 2 National Key Laboratory for Germplasm Innovation & Utilization of Horticultural Crops, College of Horticulture & Forestry Sciences, Huazhong Agricultural University, Wuhan 430070, China; wang-123@webmail.hzau.edu.cn

**Keywords:** MYC2, plant secondary metabolites, plant stress resistance, jasmonic acid, transcriptional regulation

## Abstract

Jasmonates (JAs) are essential signaling molecules that orchestrate plant responses to abiotic and biotic stresses and regulate growth and developmental processes. *MYC2*, a core transcription factor in JA signaling, plays a central role in mediating these processes through transcriptional regulation. However, the broader regulatory functions of *MYC2*, particularly in secondary metabolism and stress signaling pathways, are still not fully understood. This review broadens that perspective by detailing the signaling mechanisms and primary functions of *MYC2* transcription factors. It specifically emphasizes their roles in regulating the biosynthesis of secondary metabolites such as alkaloids, terpenes, and flavonoids, and in modulating plant responses to environmental stresses. The review further explores how *MYC2* interacts with other transcription factors and hormonal pathways to fine-tune defense mechanisms and secondary metabolite production. Finally, it discusses the potential of *MYC2* transcription factors to enhance plant metabolic productivity in agriculture, considering both their applications and limitations in managing secondary metabolite synthesis.

## 1. Introduction

MYC2 proteins are key transcription factors within the jasmonic acid (JA) signaling pathway and belong to the basic helix-loop-helix (bHLH) superfamily, with wide distribution across land plants [1]. The MYC2 protein contains a conserved bHLH domain at its C-terminus, primarily responsible for DNA binding. It targets critical *cis*-regulatory elements, including the G-box sequence (CACGTG), E-box motifs (CANNTG), and the GCG-box. Adjacent sequences rich in A/T nucleotides are also crucial, as they significantly enhance the DNA-binding affinity of these transcription factors [2,3]. MYC2 proteins can form homodimers, heterodimers, or even homotetramers through their bHLH and aspartate kinase, chorismate mutase, and tyrA (ACT) domains, enabling both activation and repression of downstream genes [4,5,6].

Furthermore, the N-terminal bHLH-MYC_N domain of MYC2 includes two critical regions: the jasmonate ZIM-domain (JAZ) interaction domain (JID) and the transcription activation domain (TAD). The JID is integral for the binding of MYC2 to JAZ proteins, which are repressors in the JA pathway, while the TAD facilitates the interaction with Mediator complex subunit 25 (MED25). A mutation from Asp128 (D128) to D128N markedly diminishes the interaction between MYC2 and JAZ proteins, although it preserves a robust interaction with MED25 [7]. Additionally, phosphorylation at this site by casein kinase II (CK2) increases the stability of MYC2 and its responsiveness to JA, while alanine substitutions at these phosphorylation sites attenuate the ability to activate JA-dependent signaling [8,9].

JAZ proteins mediate their function through three conserved domains: NT, ZIM, and Jas. The NT domain interacts with DELLA proteins, which are growth repressors in the gibberellin (GA) signaling pathway. The ZIM domain enables dimerization and engages with novel interactor of JAZ (NINJA), while the Jas domain interacts with MYC2 and coronatine insensitive 1 (COI1), facilitating JAZ degradation in response to JA signaling. Some JAZ proteins contain an EAR motif that allows direct interaction with the co-repressor TOPLESS (TPL), while others require NINJA to mediate TPL binding [10].

**Figure 1:**
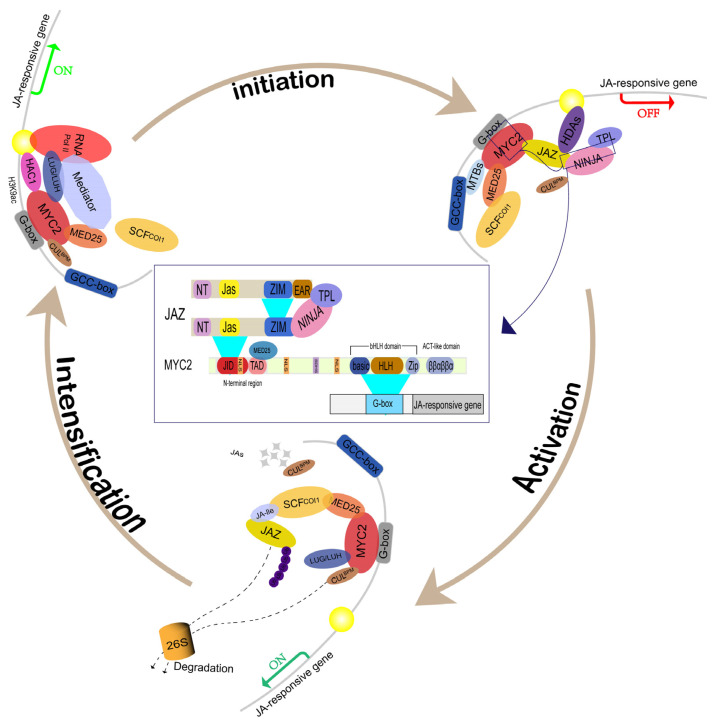
Regulation of the jasmonate signaling pathway by *MYC2* transcription factors. The dynamic regulation of the JA signaling pathway, mediated by *MYC2* transcription factors across various stages, depicts the complex interactions among key components, including MYC2, MED25, and JAZ proteins. Initialization: Interaction between MYC2 and JAZ proteins, which are characterized by NT, Jas, ZIM, and EAR domains. At this stage, JAZ proteins exert repressive functions through interactions with NINJA and TPL, effectively inhibiting MYC2 activity. Activation: Following stimulation by JA-Ile, MYC2 is released from JAZ-mediated repression. The F-box protein COI1 targets JAZ proteins for degradation, liberating MYC2 from inhibition. Subsequently, MYC2 forms a complex with MED25, recruiting RNA polymerase II and other co-activators such as HAC1 to initiate transcription of JA-responsive genes. Intensification: MED25 facilitates the recruitment of RNA Polymerase II to promoters of JA-responsive genes, supported by MYC2 binding to G-box elements. Additionally, transcription is further enhanced by the modulation of chromatin structure, specifically through acetylation of Histone 3 Lysine 9 (H3K9ac), to enable more efficient transcription. The yellow circle indicates a chromatin-associated protein complex binding site where key transcriptional regulators converge at JA-responsive promoters to mediate dynamic chromatin remodeling and gene activation or repression.

In the MYC2-JA signaling pathway (Figure 1), MED25 interacts with MYC2, COI1, JAZ, and repressors such as NINJA and TPL [11,12]. Under repressive conditions, JAZ proteins inhibit MYC2. Upon activation by JA-Ile, COI1 mediates JAZ degradation, releasing MYC2 to activate transcription [13]. MED25 enhances MYC2 function by recruiting histone acetyltransferase 1 (HAC1) and RNA polymerase II, promoting histone acetylation (H3K9) and transcription of JA-responsive genes [14]. Additionally, members of the Gro/Tup1 family genes LUG/LUH further stimulate the transcription of MYC2-regulated genes by interacting with MED25 and HAC1 [15]. Moreover, the CUL3-based E3 ubiquitin ligase (CUL3BPM) establishes a negative feedback loop that finely tunes *MYC2* expression levels during JA responses, ensuring balanced signaling [16].

In summary, *MYC2* transcription factors play a central role in regulating the JA signaling pathway, influencing crucial developmental processes, including plant growth, flowering, and responses to biotic stresses. These factors are also integral in mediating light signaling, hormonal interactions, and broader developmental aspects of plants [17,18,19]. Furthermore, MYC2 proteins play a crucial role in the biosynthesis of secondary metabolites, crucial for plant defense and survival [20,21]. Given their significant roles, this review focuses on the contributions of *MYC2* transcription factors to secondary metabolite biosynthesis, with a specific emphasis on elucidating the mechanisms through which these factors enhance plant stress resistance.

## 2. The Role of MYC2 in the Synthesis of Plant Secondary Metabolites

### 2.1. MYC2 Transcription Factors in Alkaloid Biosynthesis

**Figure 2:**
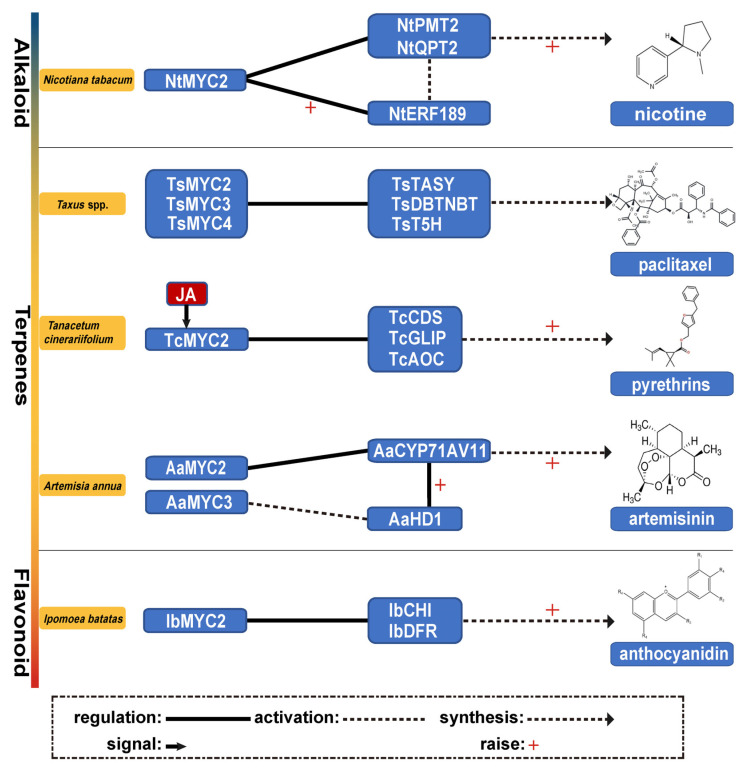
*MYC2* regulates the biosynthesis of plant secondary metabolites. *NtMYC2* controls nicotine biosynthesis in *N. tabacum* by regulating *NtPMT2*, *NtQPT2*, and *NtERF189*. *TsMYC2/3/4* modulates paclitaxel biosynthesis in *Taxus* spp. *TcMYC2*, activated by JA signaling, regulates *TcCDS*, *TcGLIP*, and *TcAOC* to drive pyrethrin synthesis in *Tanacetum cinerariifolium*. *AaMYC2* and *AaMYC3* regulate artemisinin biosynthesis in *Artemisia annua* via *AaCYP71AV1* and *AaHD1*. *IbMYC2* in *Ipomoea batatas* promotes anthocyanidin biosynthesis through *IbCHI* and *IbDFR*.

Alkaloids are nitrogen-containing secondary metabolites extensively found in plants and known for their significant biological activity. *MYC2* transcription factors regulate the expression of key enzymes and genes involved in alkaloid biosynthesis, such as methyltransferases and phosphoribosyltransferases, which play central roles in nicotine production in *Nicotiana tabacum* (Figure 2).

In tobacco, *NtMYC2* functions as a core activator of nicotine biosynthesis. *NtMYC2* binds to G-box elements within the proximal promoter regions of nicotine biosynthesis genes such as *putrescine N-methyltransferase 2* (*PMT2*) and *quinolinic acid phosphoribosyltransferase 2* (*QPT2*). It also synergistically upregulates the expression of *ERF189*, activating *PMT2* and *QPT2* [22]. Isoforms of *NtMYC2*, including *NtMYC2a*, *NtMYC2b*, and *NtMYC2c*, enhance nicotine synthesis by activating *NtPMT1a*, a gene encoding a rate-limiting enzyme in the pathway. The activity of *NtMYC2* is also influenced by its interactions with JAZ repressor proteins [23]. CRISPR-Cas9 knockout of *NtMYC2a* in tobacco has shown a dramatic decrease in the expression of nicotine biosynthetic and transport genes, resulting in about an 80% reduction in nicotine levels in the leaves [24]. These findings confirm the essential role of *NtMYC2a* in regulating alkaloid biosynthesis and highlight its potential as a key target for metabolic engineering. Additional studies reveal that *NtMYC2a*-knockout tobacco plants display narrower leaves and increased accumulation of nor-nicotine and methylamine [25], suggesting a broader impact of *NtMYC2a* on secondary metabolic pathways.

In *Catharanthus roseus*, *CrMYC2* plays a significant role in early JA responses, controlling the expression of terpenoid indole alkaloid (TIA) biosynthetic genes. *CrMYC2* specifically targets *AP2* domain genes *ORCA2*, *ORCA3*, and *ORCA4* by binding to G-box-like elements in their promoters, activating these genes and enhancing TIA production [26,27]. In addition to its activating role, *CrMYC2* is also involved in a regulatory network that includes repressive factors. *G-box binding factors 1* (*CrGBF1*) and *CrGBF2* function as negative regulators of TIA biosynthesis by competing with *CrMYC2* for binding to promoter regions of target genes, thereby limiting *MYC2*-mediated transcriptional activation [28]. Additionally, *CrbHLH05*, also known as *repressor of MYC2 targets 1* (*CrRMT1*), does not dimerize with *CrMYC2* but competes for binding to the T/G-box in the *ORCA3* promoter, acting as a passive repressor that antagonizes the activity of *CrMYC2* on its targets [29].

In tomato, *SlMYC1* and *SlMYC2* play significant roles in the production of phenylpropanoid–polyamine conjugates and steroidal glycoalkaloids (SGAs) through the JA signaling pathway. Double knockout of *SlMYC1* and *SlMYC2* in hairy roots results in dramatically reduced basal expression of SGA biosynthetic genes and significant decreases in α-tomatine and dehydrotomatine content [30]. In addition to their role in the transcriptional regulation of SGA biosynthesis, *SlMYC2* also mediates JA–GA crosstalk by modulating the expression of GA catabolic genes, thereby influencing growth–defense trade-offs. JA deficiency suppresses SGA accumulation, whereas low GA levels or impaired GA signaling enhance SGA production [31]. In *Lycoris aurea*, *LaMYC2* binds to E-box elements in the promoter of *tyrosine decarboxylase* (*LaTYDC*), a gene involved in lycorine biosynthesis, acting as a positive regulator of this Amaryllidaceae alkaloid [32].

### 2.2. MYC2 Transcription Factors in Terpenoid Biosynthesis

Terpenoids, a diverse class of compounds including monoterpenes, sesquiterpenes, diterpenes, triterpenes, and polyterpenes, play important roles as volatile and non-volatile secondary metabolites in plants. *MYC2* transcription factors serve as pivotal regulators of terpenoid biosynthesis by modulating the expression of key biosynthetic genes.

Monoterpenes, such as pyrethrins derived from the flowers of *Tanacetum cinerariifolium*, are effective broad-spectrum insecticides that are safe for mammals and widely used in organic agriculture and household pest control [33]. The *TcMYC2* gene in *T. cinerariifolium* responds to JA treatment, directly regulating pyrethrin synthesis by binding to the promoters of biosynthetic genes such as *chysanthemyl diphosphate synthase* (*TcCDS*), GDSL *lipase-like acyltransferase* (*TcGLIP*), and *allene oxide cyclase* (*TcAOC*) at E/G-box elements [34]. Another MYC2-like transcription factor, *TcbHLH14*, also targets *TcCDS* and *TcAOC* but not *TcGLIP*, resulting in only a moderate increase in pyrethrin levels [35].

Linalool is a major component of volatiles that plays a crucial role in plant fragrance and pollination. In *Osmanthus fragrans*, *OfMYC2* promotes linalool biosynthesis by transcriptionally activating *OfTPS2* in cooperation with *OfMYB21*, although this activation is suppressed by *OfJAZ3* through its interaction with *OfMYC2* [36]. In *Chimonanthus praecox*, the expression of *CpMYC2* correlates with flowering stages, showing a significant increase from bud to full bloom. Overexpression of *CpMYC2* in *Arabidopsis* and tobacco significantly enhances the production of volatile monoterpenes, especially linalool. *CpMYC2* interacts with the JA signaling pathway to upregulate *TPS* gene expression in response to JA and GA treatments, increasing the release of aromatic compounds [37]. In *Freesia hybrida*, linalool synthesis is predominantly mediated by the monoterpene synthase *TPS1*. *FhMYC2* acts as a negative regulator by restricting *FhMYB21* binding to the *FhTPS1* promoter, inhibiting linalool synthesis. In contrast, co-expression of *AtMYC2* with *AtMYB21* in *Arabidopsis thaliana* preferentially activates sesquiterpene genes while suppressing the monoterpene gene *AtTPS14*, favoring sesquiterpene over monoterpene emission, including linalool [38]. Additionally, *FhMYB108* in *F. hybrida* enhances linalool synthesis by promoting *FhTPS1* expression. Interestingly, interactions between *FhMYC2* and *FhMYB108* proteins in *Arabidopsis* inhibit *AtTPS14* activation, revealing the complex molecular mechanisms by which *MYC2* collaborates with *MYB* transcription factors to regulate volatile terpenoid biosynthesis [39].

Sesquiterpenes play significant roles in plant defense and pollinator attraction [40,41]. In sweet orange, *CitMYC3*, a *MYC2* homolog, functions as a key regulator of valencene synthesis, activating the promoter and enhancing the expression of the sesquiterpene synthase gene *CsTPS1* [42]. In *A. thaliana*, *AtMYC2* controls sesquiterpene production by binding to the promoters of *TPS21* and *TPS11*, thus stimulating the expression of these genes and facilitating the release of compounds such as (*E*)-β-caryophyllene, which is influenced by both GA and JA [43].

Artemisinin, a sesquiterpene lactone from *Artemisia annua*, is synthesized in glandular cells [44]. The JA-responsive transcription factor *AaMYC2* enhances artemisinin production by binding to the promoters of key artemisinin biosynthesis genes *CYP71AV1* and artemisinic aldehyde *double bond reductase* (*DBR2*). Transgenic plants overexpressing *AaMYC2* show elevated levels of artemisinin [45]. Additionally, *AaMYC2*, together with *AabHLH1*, responds to both MeJA and abscisic acid (ABA), activating crucial genes involved in artemisinin synthesis and thus boosting its yield [46]. The *MYC2* homolog *AaMYC3* also plays a significant role by regulating glandular density and artemisinin biosynthesis. Overexpression of *AaMYC3* results in increased glandular density and artemisinin content, while RNA interference targeting *AaMYC3* decreases both. *AaMYC3* activates the transcription of *AaHD1*, which is involved in gland development, and upregulates artemisinin biosynthetic genes, including *CYP71AV1* and aldehyde dehydrogenase 1 (*ALDH1*). It further collaborates as a co-activator with *AabHLH1* and *AabHLH113* to enhance the transcription of key artemisinin biosynthesis genes, *amorpha*-4,11-*diene synthase* (*ADS*) and *DBR2*, thereby amplifying artemisinin production [47]. Contrarily, *AabHLH2* and *AabHLH3* act as transcriptional repressors by competing for the same *cis*-regulatory elements as *AaMYC2*, albeit lacking a conserved activation domain, which suggests they inhibit artemisinin synthesis [48].

Sesquiterpenes derived from agarwood are recognized for their antibacterial and antifungal properties and typically accumulate in response to elicitation or wounding signals. In *Aquilaria sinensis*, *AsMYC2* is repressed by AsJAZ1 repressor protein, thereby preventing it from activating the expression of *agarwood sesquiterpene synthase gene 1* (*ASS1*). Under normal conditions, *ASS1* exhibits low expression levels, resulting in limited sesquiterpene production. However, in wounded *A. sinensis*, endogenous JA biosynthesis triggers the release of *AsMYC2*, which directly targets and activates *ASS1*, enhancing sesquiterpene biosynthesis [49]. In *Gossypium hirsutum*, the sesquiterpene aldehyde gossypol acts as a crucial antimicrobial metabolite for defense against pathogens and insect predation. *GhMYC2* influences the gossypol biosynthetic pathway by regulating the activity of enzymes in the cytochrome P450 (CYP450) superfamily, specifically *CYP71BE79*, modulating gossypol production [50].

In *Lavandula angustifolia*, overexpression of *MYC2* homolog *LaMYC4* is associated with increased levels of sesquiterpenes, including caryophyllenes, in *Arabidopsis* and tobacco. This overexpression correlates with enhanced gene activity, which is crucial for terpenoid biosynthesis and increases the number and size of glandular trichomes [51]. In *Curcuma wenyujin*, *CwMYC2*, a key regulator within the JA signaling pathway, significantly upregulates genes associated with β-elemene biosynthesis, thereby enhancing β-elemene accumulation in the leaves [52]. In *Oryza sativa*, *OsMYC2* promotes the biosynthesis of resistance compounds, including the monoterpene geraniol and the sesquiterpene caryophyllene [53].

Diterpenes are pivotal in plant defense mechanisms and valuable in human medicine. Paclitaxel, a crucial anticancer drug used to treat ovarian and breast cancers, is synthesized under strong induction by JA [54]. In *Taxus* species, transcription factors such as *MYC2*, *MYC3*, and *MYC4* regulate essential genes in the paclitaxel biosynthetic pathway, including *taxane synthase* (*TASY*), *10-deacetylbaccatin III-10β-O-acetyltransferase* (*DBTNBT*), and *taxadiene 5-alpha hydroxylase* (*T5H*). These transcription factors bind to the promoters of these genes, activating their transcription and thus promoting paclitaxel production [55]. In *Taxus chinensis*, the transcription factor *TcMYC2a* directly binds to the promoter of the *TASY* gene, enhancing *TASY* expression and potentially influencing paclitaxel biosynthesis by upregulating *TcERF15* [56].

Tanshinones, representative diterpenoid compounds in *Salvia miltiorrhiza*, are synthesized through a pathway that involves key enzymes such as *Hydroxycinnamate-CoA ligase 6* (*SmHCT6*) and P450 monooxygenase (*SmCYP98A14*), which are induced by JA. *SmMYC2a* activates the expression of both *SmHCT6* and *SmCYP98A14* by binding to the E-box elements in their promoters, while *SmMYC2b* specifically regulates *SmCYP98A14* [57]. *SmMYC2* collaborates with *SmbHLHL37* to enhance the transcription of genes such as geranylgeranyl pyrophosphate synthase (*SmGGPPS*), thereby promoting tanshinone synthesis. It also dynamically interacts with *SmMYB36* to regulate JA-mediated tanshinone accumulation [58,59]. Conversely, *SmbHLH60* acts as a negative regulator, competing with *SmMYC2* for control and repressing transcription of targets such as *tyrosine aminotransferase 1* (*SmTAT1*) and *dihydroflavonol 4-reductase* (*SmDFR*), thus inhibiting phenolic acid and anthocyanin biosynthesis pathways in *S. miltiorrhiza* [60]. In *Salvia sclarea*, overexpression of *A. thaliana AtMYC2* and *AtWRKY40* in hairy roots activates the methylerythritol 4-phosphate pathway, enhancing diterpene content [61]. Heterologous expression of *AtMYC2* in *S. miltiorrhiza* hairy roots significantly upregulates genes including *1-deoxy-D-xylulose 5-phosphate synthase* (*SmDXS2*) and *SmTAT*, leading to enhanced accumulation of tanshinones and phenolic acids, with tanshinone yield reaching 14.06 mg/g dry weight—5.45 times that of the control—and phenolic acid yield at 95.9 mg/g dry weight, a 3.3-fold increase [62].

In *Tripterygium wilfordii*, *TwMYC2a* and *TwMYC2b* negatively regulate triptolide biosynthesis by inhibiting the expression of *TwTPS27a* and *TwTPS27b* in hairy roots [63]. In *Ginkgo biloba*, *GbMYC2* binds directly to the G-box in the promoter of the *levopimaradiene synthase* (*GbLPS*) gene, activating genes associated with ginkgolide biosynthesis [64]. Furthermore, *GbMYC2* enhances ginkgolide biosynthesis by activating the expression of the *GbGGPPS* gene through promoter binding. *GbMYC2_4* selectively binds to the canonical G-box motif within the *GbGGPPS* promoter, while *GbMYC2_5* preferentially interacts with an adjacent A/T-rich G-box-like motif, thereby synergistically activating *GbGGPPS* expression [65].

Carotenoids, tetraterpenoid compounds responsible for the vibrant coloration in citrus fruits, are influenced by the red carotenoid β-citraurin, a key pigment in the peel of the Newhall orange. MeJA treatment significantly enhances β-citraurin production and coloration. This induction upregulates *CsMYC2*, which then activates the gene *CsCCD4b* in the β-citraurin biosynthetic pathway by binding to its promoter, thereby impacting fruit coloration. Additionally, *CsMYC2* promotes the expression of *CsMPK6*, which interacts with *CsMYC2*, reducing its stability and DNA-binding activity, thus establishing a negative feedback loop that modulates JA signaling during fruit coloration [66]. In *Chrysanthemum indicum* var. aromaticum, MeJA induces the expression of *CiMYC2*, whose overexpression in tobacco leads to shorter plants, deeper leaf color, increased chlorophyll and carotenoid content, and the production of new terpenoid compounds [67].

In the rubber tree (*Hevea brasiliensis*), the small rubber particle protein (SRPP) plays a key role in natural rubber biosynthesis. *HbMYC2b*, highly expressed in the bark, binds to the *HbSRPP* promoter to activate its transcription, thus positively regulating *HbSRPP* expression [68]. In *Taraxacum kok-saghyz*, overexpression of *TkMYC2* not only inhibits leaf development and promotes root growth but also enhances natural rubber production. *TkSRPP* and *rubber elongation factor* (*TkREF*) genes are upregulated in transgenic lines, suggesting that *TkMYC2* regulates natural rubber synthesis by modulating *TkSRPP/REF* expression [69].

In *Gynostemma pentaphyllum*, *GpMYC2* plays a critical role in activating the synthesis of gypenosides by binding to the promoters of genes involved in their biosynthesis, thereby confirming the significant role of the COI1/JAZ/MYC2 module in regulating responses induced by MeJA [70]. *Taraxacum antungense*, a traditional herb known for its antibacterial and antioxidant properties and rich triterpenoid content, also demonstrates the regulatory capabilities of MeJA. The overexpression of *TaMYC2*, induced by MeJA, significantly enhances triterpenoid accumulation, with the expression level of the *squalene synthase gene* (*TaSS*) elevated three to five times compared to control lines [71]. In *Bupleurum chinense*, *BcMYC2* promotes the expression of key enzyme genes such as *3-Hydroxy-3-methylglutaryl-coenzyme A reductase* (*HMGR*), *isopentenyl diphosphate isomerase* (*IPPI*), *farnesyl pyrophosphate synthase* (*FPS*), and p-acetoxydihydrochalcone synthase (*p-AS*), which are essential for the saikosaponin biosynthetic pathway [72].

### 2.3. MYC2 Transcription Factors in Flavonoid Synthesis

Flavonoids, recognized for their potent antioxidant properties, are key plant secondary metabolites abundant in fruits, vegetables, and flowers. In *A. thaliana*, the *MYC2* homolog *MYC3* plays a leading role in enhancing plant resistance to insect herbivory. Mutants of *MYC3* show significantly reduced levels of flavonoid compounds, potentially alleviating wound-induced growth suppression [73]. In apples, *MdMYC2*, responsive to JA, significantly upregulates genes associated with anthocyanin synthesis and effectively increases anthocyanin content [74]. The regulation of anthocyanins by *MYC2* is complex, exhibiting both activation and repression. *MYC2* can activate structural genes in the anthocyanin biosynthetic pathway. However, in the presence of the MYB/bHLH/WD40 complex, MYC2 binds to GL3, a bHLH component of the complex, thereby inhibiting its formation and repressing anthocyanin synthesis. This dual regulatory role is further influenced by interactions with negative regulators such as *BBX21* and *SPL9* [75]. In rice, *OsMYC2* enhances the expression of *naringenin 7-O-methyltransferase* (*OsNOMT*), a key gene in sakuranetin production, by activating its promoter in response to JA treatment. This process is further amplified through interactions with *OsMYC2* homologs, *OsMYL1* and *OsMYL2*, collectively promoting JA-induced sakuranetin synthesis [76].

In *Ipomoea batatas*, MYC2 plays a crucial role in enhancing anthocyanin accumulation by binding to promoters of key biosynthetic genes such as *IbCHI* and *IbDFR*. Overexpression of *IbMYC2* under saline and drought conditions enhances the expression of genes related to reactive oxygen species (ROS) scavenging and proline synthesis, increasing stress tolerance [77]. In *Vitis vinifera*, the biosynthesis of flavonols, anthocyanins, and proanthocyanidins (PAs) is distinctly regulated both temporally and spatially during berry development, co-regulated by MYC and MYB transcription factors. *VvMYC2* interacts with *MYB24* to co-regulate the synthesis of terpenes and flavonols under UV and high-intensity visible light stress in grape skin regions devoid of anthocyanins, enhancing fruit tolerance to light stress [78].

In *Medicago sativa*, the expression of *MsMYC2* is significantly upregulated in the *mtugt84a1* mutant, where the UDP-glycosyltransferase suppresses the JA signaling pathway through glycosylation and feedback regulation, impacting anthocyanin accumulation [79]. In *Zea mays*, overexpression of *ZmMYC2* in *Arabidopsis* mutants restores JA sensitivity, leading to inhibited root growth and increased anthocyanin accumulation [80]. In *Camellia sinensis*, *CsMYC2* positively regulates flavan-3-ol biosynthesis under JA signaling. However, splice variants CsJAZ1-1, CsJAZ1-2, and CsJAZ1-3 form complexes with CsMYC2, suppressing the expression of key flavan-3-ol biosynthetic genes [81].

### 2.4. Regulatory Role of MYC2 in VOCs, Essential Oils, and Other Compounds

In *Litsea cubeba*, an essential oil-rich plant valued for its high neral and geranial content (up to 80% of oil composition), *LcMYC2* enhances essential oil biosynthesis by binding to the promoters of *LcTPS42* and *LcGPPS* [82]. In *A. thaliana*, (*E*)-2-hexenal, a key compound involved in plant communication and pest resistance, induces the expression of *WRKY46* and *MYC2*. Together, these factors activate *respiratory burst oxidase homolog d* (RBOHD) and flavonoid biosynthesis genes, thereby increasing total flavonoid accumulation and enhancing pest resistance [83].

In Chinese cabbage (*Brassica rapa* ssp. pekinensis), *BrMYC2* overexpression significantly enhances the accumulation of glucosinolate (GS), compounds that confer strong resistance to bacterial soft rot [84]. In callus tissues of apple, *MdMYC2* directly binds to the promoter of *MdLOX5a*, significantly enhancing the biosynthesis of volatile aldehydes and alcohols essential for flavor and aroma [85].

## 3. Response of MYC2 to Stress

**Figure 3:**
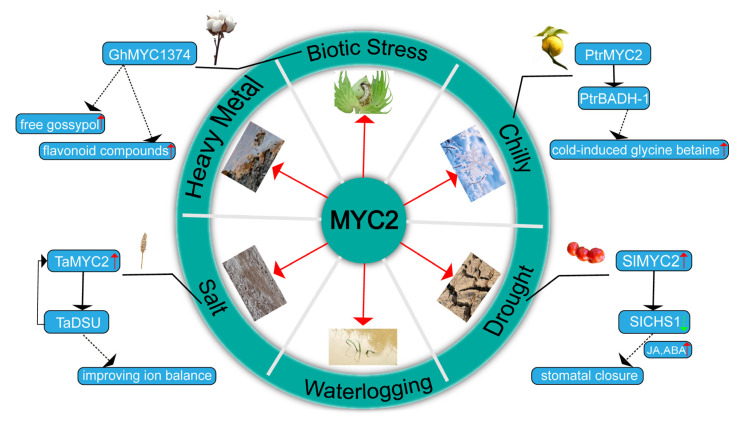
Overview of *MYC2* transcription factors’ role in plant stress response pathways. *MYC2* transcription factors regulate plant responses to a wide range of biotic and abiotic stresses, including cold, drought, salinity, and heavy metals toxicity. They play key roles in enhancing stress tolerance through diverse mechanisms. Red arrows indicate positive regulation, green arrows represent downregulation, black solid arrows show gene regulation, and black dashed arrows indicate phenotypic or substance changes. For example, *PtrMYC2* promotes glycine betaine accumulation to improve cold tolerance; *SlMYC2* facilitates stomatal closure under drought conditions; *TaMYC2* helps maintain ion balance during salt stress; and *GhMYC1374* induces flavonoid and gossypol biosynthesis in response to biotic stress.

MYC2 plays a significant role in regulating the synthesis of plant secondary metabolites and acts as a key regulator in plant responses to abiotic stresses. It activates downstream stress-responsive genes, enhancing plant adaptation to cold, drought, salinity, and other adverse conditions. An overview of *MYC2*-mediated stress response pathways is illustrated in Figure 3.

### 3.1. The Role of MYC2 in Cold Stress

Plants enhance cold tolerance primarily through the activation of the *C-repeat-binding factor* (*CBF*) pathway. Under non-stress conditions, JAZ1/4 interacts with *inducer of CBF expression 1* (ICE1) and ICE2 to suppress this pathway. Upon exposure to cold, JA biosynthesis is induced, leading to the degradation of JAZ1/4 proteins. This relieves repression of the ICE-CBF cascade and allows activation of downstream cold-responsive genes. *MdMYC2* enhances frost resistance by directly regulating the expression of *MdCBF1*, thereby improving cold tolerance in apples [86]. In *Manihot esculenta*, *MeMYC2.2* is significantly upregulated under cold stress. It activates *MeCBF3* expression, and its overexpression in transgenic *Arabidopsis* plants leads to enhanced cold tolerance [87]. In peach fruits, exogenous MeJA treatment upregulates *PpMYC2.2*, activating the JA signaling pathway, reducing electrolyte leakage, and protecting cell membranes by regulating lipid metabolism. *PpMYC2.2* also synergizes with the CBF pathway, particularly *PpCBF3*, to increase peach cold tolerance [88]. Similarly, in the winter wheat variety Dn1, which is capable of successfully overwintering at extremely low temperatures, expression of *TaMYC2* is induced by cold stress and JA treatment. This activation triggers the ICE-CBF-COR cold resistance pathway, significantly enhancing cold tolerance. At freezing temperatures, cell lines overexpressing *TaMYC2* exhibit reduced electrolyte leakage, lower malondialdehyde content, increased proline levels, and enhanced antioxidant defenses [89].

Beyond these examples, additional studies in other species have further highlighted the central role of *MYC2* in cold stress responses. In cold-exposed *Poncirus trifoliata*, the *betaine aldehyde dehydrogenase gene* (*PtrBADH-1*) is activated by *PtrMYC2*, regulating the accumulation of cold-induced glycine betaine, which helps the plant to cope with low-temperature stress [90]. In *Trifolium ambiguum*, *TaMYC2* is responsive to multiple stresses, including salt, alkali, cold, and drought, and is induced by plant hormones such as IAA, GA3, and MeJA. Cold and drought stresses specifically induce *TaMYC2* expression, further enhancing the activity of antioxidant enzymes [91].

In tomato, *SlMYC2* enhances cold tolerance by modulating polyamine biosynthesis and oxidative stress management. This gene upregulates *arginine decarboxylase 1 (ADC1*), leading to increased putrescine production, which decreases ROS levels and alleviates oxidative stress [92]. Treatment with MeJA boosts the activities of the ascorbate–glutathione (AsA-GSH) cycle enzymes and activates the SlICE-SlCBF-SlCOR signaling pathway, which is critical for cold damage mitigation. Silencing *SlMYC2* curtails these protective effects, underscoring its crucial role in these pathways [93]. *SlMYC2* also triggers the expression of *glutathione S-transferase U24* (*SlGSTU24*) and *β-Amylase 3* (*SlBAM3*), facilitating ROS reduction and starch degradation, respectively, thus improving cold tolerance [94,95]. Moreover, *MYC2* enhances the expression of nine-*cis*-epoxycarotenoid dioxygenase 2 (*NCED2*), increasing ABA accumulation and strengthening plant tolerance to low-temperature stress [96]. Simultaneously, *MYC2* activates *SlERF.B8*. This gene not only responds to cold stress and JA signals but also forms a positive feedback loop with MYC2, amplifying JA signaling and further enhancing the plant’s cold tolerance [97]. Additionally, *SlMYC2* binds to G/E-box elements to activate genes like *arginase* (*SlARG1* and *SlARG2*), *arginine decarboxylase* (*SlADC*), and *ornithine decarboxylase* (*SlODC*), boosting polyamine levels and further mitigating cold stress [98]. Furthermore, *SlMYC2* is integral to the crosstalk between JA and melatonin (MT) pathways. Cold stress induces JA accumulation, which upregulates MYC2-activated genes involved in MT biosynthesis, including Serotonin N-acetyltransferase (*SlSNAT*) and *acetyl-5-hydroxytryptamine O-methyltransferase* (*SlAMT*). Increased MT accumulation not only potentiates cold tolerance but also promotes further JA biosynthesis, creating a positive feedback loop that amplifies cold responses [99].

### 3.2. The Role of MYC2 in Drought Tolerance

*MYC2* transcription factors play a significant role in plant drought tolerance by modulating diverse drought-responsive pathways. In *A. thaliana*, *AtMYC2* enhances drought resistance by directly binding to the promoter of the *early responsive to dehydration 1* (*ERD1*) gene, a critical player in drought adaptation [100].

In tomato, *SlMYC2* improves drought tolerance through multiple regulatory routes. It represses *protein phosphatase 2C1* (*SlPP2C1*) and the cytokinin signaling component *SlRR26*, negatively affecting ROS accumulation and stomatal closure. Overexpression of *SlRR26* reduces drought tolerance, whereas *slrr26* mutants show enhanced resistance [101]. Moreover, *SlMYC2* represses *chalcone synthase 1* (*SlCHS1*), decreasing flavonol levels and increasing ROS in guard cells. This promotes stomatal closure by elevating JA and ABA levels [102].

Similar regulatory functions of *MYC2* have been observed in other crops. In *Brassica napus*, silencing *BnMYC2* disrupts stomatal closure under light and dark conditions, resulting in excessive water loss and decreased drought tolerance [103]. In sorghum, *SbMYC2* is strongly induced by polyethylene glycol (PEG)-simulated drought and JA treatment. Its overexpression enhances drought resistance in *Arabidopsis*, rice, and sorghum by reducing ROS levels and maintaining higher chlorophyll content [104].

In wheat, *MYC2* co-regulates melatonin biosynthesis by directly interacting with the promoter of the *N-acetylserotonin methyltransferase* (*ASMT*) gene. This MYC2-ASMT module enhances drought tolerance by modulating melatonin levels in wheat leaves [105]. Additionally, the miR1119-MYC2 regulatory module has been identified in wheat roots, where it influences ABA hormone levels, water relations, and photosynthetic activity, enhancing drought tolerance through hormonal crosstalk [106]. In poplar, *MYC2* regulates stomatal development by targeting the promoters of stomatal density-related genes, including *epidermal patterning factor 2* (*PpnEPF2*), *PpnEPFL4*, and *PpnEPFL9*. Overexpression of *PpnMYC2* in both poplar and *Arabidopsis* results in reduced stomatal density, improved water use efficiency, and enhanced drought resistance [107]. In barley (*Hordeum vulgare*), MYC2 directly binds to the JA response element in the promoter of *Ribulose*-1,5-*bisphosphate carboxylase/oxygenase activase A* (*RcaA*), enhancing photosynthetic efficiency under combined drought and salt stress. This regulatory module improves plant water status [108].

### 3.3. The Role of MYC2 in Water Stress

Environmental water stress, including rainfall, submersion, and osmotic stress, triggers short-term molecular responses and long-term developmental adjustments in plants. *MYC2* transcription factors play a central role in regulating these responses by integrating hormonal and oxidative stress signaling. Simulated rainfall, through water spray stress, activates the JA signaling pathway. In this process, *MYC2* interacts with *bHLH19* and *ERF109* to activate *octadecanoid-responsive AP2/ERF-domain 47* (*ORA47*), promoting JA biosynthesis and forming a positive feedback loop that enhances JA accumulation [109]. In *C. sinensis*, *MYC2* activates the transcription of JA biosynthesis-related genes and *peroxidase* (*PER*) genes by binding to their promoters, forming a positive feedback loop that enhances tea plant tolerance to osmotic stress [110]. Similarly, in sunflowers, *MYC2* integrates JA and ABA signaling pathways, activating stress-responsive transcription factors such as *dehydration 20* (*RD20*), *RD22*, *RD26*, *ANAC19*, and *ANAC29*. Concurrently, the JA and SA pathways jointly activate the *WRKY70* transcription factor, enhancing plant tolerance to water stress [111]. In *A. thaliana*, *MYC2* interacts with *MYB30* to regulate the expression of antioxidant genes, including *Vitamin C defective 1* (*VTC1*) and *Glutathione synthetase 1* (*GSH1*). This interaction integrates light signals with reoxygenation stress responses, enhancing the plant’s antioxidant capacity and improving survival rates. Overexpression of *VTC1* and *GSH1* can completely rescue the hypersensitivity to submersion observed in *myc2* mutants [112].

Although these studies differ in experimental systems, stress types, and target genes, they consistently support the notion that *MYC2* acts as a central regulatory hub in plant water stress responses. Apparent differences in downstream mechanisms likely reflect species-specific signaling networks and stress-specific regulatory crosstalk.

### 3.4. Role of MYC2 in Salt Stress

The role of MYC2 transcription factors in response to salt stress is complex, exhibiting both positive and negative regulatory effects across different plant species. Within the JA signaling pathway, *MYC2* genes generally increase plant sensitivity to salt. For instance, in *Arabidopsis*, JA suppresses the expression of the antioxidant enzyme *Catalase 2* (CAT2) through *MYC2*, reducing seedling salt tolerance [113]. Additionally, *MYC2* binds to the 5-UTR of the key rate-limiting enzyme *delta1-pyrroline-5-carboxylate synthase* (*P5CS1*) in proline biosynthesis, negatively regulating proline synthesis and further diminishing the plant salt tolerance [114]. In *Curcuma wenyujin*, the CwJAZ4/9 complex inhibits the JA-induced terpene synthesis pathway by interacting with *CwMYC2*, leading to reduced terpenoid accumulation but enhancing the salt tolerance of hairy roots, maintaining their growth under salt stress [115].

Conversely, *MYC2* genes positively regulate salt stress tolerance in some plants, particularly through ABA-related pathways. In *S. miltiorrhiza*, overexpression of *SmMYC2* boosts salt tolerance by increasing the activities of antioxidant enzymes (SOD, POD, and CAT) and proline content. This upregulation also involves the activation of flavonoid biosynthesis genes, further improving the plant’s antioxidant capacity and salt stress tolerance [116]. Similarly, in wheat, the *small ubiquitin-like modifier (SUMO) protease gene* (*TaDSU*) interacts with *MYC2*, reducing its sumoylation levels and enhancing its transcriptional activity. This interaction establishes a positive feedback loop, where *MYC2* binds to the *TaDSU* promoter, boosting its expression and improving ion balance (higher K^+^/Na^+^ ratio) and salt tolerance [117]. In *Caragana korshinskii*, *CkMYC2* regulates the expression of the *pyrabactin resistance like 4* (*CkPYL4*) gene, promoting ABA accumulation in roots and thereby enhancing plant tolerance to both salt and drought stress [118]. Additionally, in rice, a MYC2-like transcription factor binds to the ABA-responsive element in the promoter of *cytochrome P450 family 2* (*OsCYP2*), enhancing salt tolerance, partially restoring the salt tolerance of cyp2-RNAi rice, and increasing antioxidant enzyme activity [119].

### 3.5. Role of MYC2 in Heavy Metal Stress

Reports on the role of *MYC2* transcription factors in heavy metal stress are limited. In *A. thaliana*, *MYC2* suppresses the expression of *heavy metal ATPase gene 2* (*HMA2*) and *HMA4*, altering cadmium (Cd) distribution and reducing tolerance. However, MYC2 degradation under Cd stress partially alleviates its repressive effects, aiding stress adaptation [120]. In wheat, *TaMYC8* negatively regulates Cd-responsive ethylene signaling. *TabHLH094*, a Cd-induced bHLH transcription factor, inhibits *TaMYC8* activity, reducing its binding to the *TaERF6* promoter and limiting ethylene biosynthesis. Overexpression of *TabHLH094* enhances wheat Cd tolerance by modulating TaMYC8 activity and suppressing ethylene production [121].

### 3.6. Role of MYC2 in Biotic Stress

*MYC2* transcription factors play significant roles in enhancing plant biotic stress. In tomatoes, the *Solyc08g005050*, which belongs to the *MYC2* subfamily, interacts synergistically with the HD-ZIP IV transcription factor (*Wo*) to promote trichome development and terpene biosynthesis, thereby enhancing resistance to spider mites [122]. Similarly, in chrysanthemums, *CmMYC2* regulates the development of T-shaped and glandular trichomes and the accumulation of terpenoid compounds. By directly activating *CmMYBML1* and forming a feedback inhibition loop with it, while *CmMYC2* promotes the initial activation of *CmMYBML1*, the overexpression of *CmMYBML1* subsequently binds and consumes *CmMYC2*, preventing sustained activation of *CmMYBML1*. These mechanisms significantly enhance the plant’s resistance to herbivorous larvae [123]. The *MYC2-like transcription factor pigment gland formation* (*GoPGF*) activates the expression of jasmonate-associated *VQ motif-like protein* (*JAVL*) in cotton, which establishes a negative feedback loop by inhibiting the transcription of *GoPGF*, thereby balancing *GoPGF* and *JAVL* expression. Furthermore, *JAVL* regulates JA levels by inhibiting the expression of JA synthesis-related genes through its interaction with *GoPGF*. An increase in the *GoPGF* to *JAVL* expression ratio leads to enlarged pigment glands and increased accumulation of JA and defense compounds, thereby enhancing cotton’s resistance to insects and pathogens [124]. In cotton, the MYC2-like transcription factor *GhMYC1374* is significantly induced under aphid attack. Studies indicate that *GhMYC1374* enhances cotton’s resistance to aphids by activating the biosynthesis of flavonoid compounds and free gossypol. The overexpression of *GhMYC1374* significantly increases cotton’s resistance to aphids, while silencing *GhMYC1374* via VIGS technology reduces its resistance [125].

In *Arabidopsis*, *MYC2* induces the expression of the cell wall acetylation gene *trichome birefringence-like 37* (*AtTBL37*), enhancing cell wall acetylation, thereby strengthening resistance to herbivores [126]. In *B. napus*, MeJA-induced *BnMYC2* positively regulates the expression of the anti-insect gene *vegetative storage protein 2* (*VSP2*), enhancing the plant defense against insect stress [127]. In tomatoes, *SlMYC2* regulates the *MeJA-induced gene* (*SlJIG*) by directly binding to its promoter, leading to the activation of *TPS* genes and enhanced resistance to insect and microbial stress [128].

In rice, *MYC2* triggers transcriptional cascades by regulating secondary transcription factors like *bHLH6*, amplifying JA responses across tissues. Additionally, *MYC2* establishes a feedback mechanism by modulating the expression of JA repressors and catabolic genes, including *NAC* transcription factors (*NAC1*, *NAC3*, and *NAC4*), which attenuate JA responses and reduce defense capabilities against insect herbivores [129]. In tomato, *MYC2* activates the *E3 ubiquitin ligase* (*PUB22*), facilitating JAZ protein degradation via the 26S proteasome pathway. This mechanism strengthens defense against *Helicoverpa armigera*. The MYC2-PUB22-JAZ4 module also regulates JA-mediated responses, such as resistance to *Botrytis cinerea*, inhibition of root elongation, and anthocyanin accumulation [130].

In *C. sinensis*, *CsMYC2.2* directly binds to the promoter of *CsGSTU45*, activating its expression and reducing resistance to *Colletotrichum camelliae*. Silencing *CsMYC2.2* significantly enhances resistance to tea cake disease [131]. In maize, *ZmMYC7* activates *ZmERF147* and defense genes like *pathogenesis-related protein 1* (*ZmPR1*), *ZmPR2*, and *ZmPR3*, bolstering resistance to *Fusarium graminearum* and *Setosphaeria turcica* [132]. Furthermore, *ZmMYC2a* and *ZmMYC2b* enhance the synthesis of defense metabolites such as benzoxazinoids and volatile terpenes in response to JA signaling or simulated herbivory. Double mutant studies reveal increased insect susceptibility and reduced production of these metabolites [133].

### 3.7. MYC2 Mediators of Hormonal Interactions and Stress Adaptation in Plants

*MYC2* transcription factors are central players in JA signaling, integrating multiple hormonal pathways. In the JA-ABA pathway, *MYC2* interacts with *ABA-insensitive 5* (*ABI5*) and directly activates the *ABA2* promoter through the MKK3-MPK6-MYC2 module, enhancing ABA biosynthesis and modulating ABA-mediated responses such as drought responses and seed germination inhibition [134,135].

In JA-SA crosstalk, *MYC2* plays dual regulatory roles. It interacts with SA-induced *NPR1*, which inhibits JA-responsive genes by forming complexes with *MYC2* and blocking its interaction with MED25 [136]. Simultaneously, *MYC2* promotes SA biosynthesis and signaling, enhancing defenses like PAMP-triggered immunity (PTI) and effector-triggered immunity (ETI) [137]. This JA-SA division of labor is evident in plant defense against chewing and piercing-sucking insects, where JA enhances resistance to caterpillars while inhibiting whiteflies [138].

In JA-GA crosstalk, *MYC2* regulates SGA biosynthesis and GA catabolism by activating GA metabolic genes such as *GA2ox3* and *GA2ox7*, which inactivate GA and redistribute resources under stress conditions, including herbivore attacks like those from *Nilaparvata lugens*. Additionally, the DELLA interacts with MYC2, disrupting JA regulation of SGA metabolism and affecting *MYC2* activity [31,139].

This multi-hormonal cross-regulatory network not only optimizes plant adaptability to environmental conditions but also enhances survival strategies under complex adversities [140,141].

## 4. Applications and Prospects of MYC2 Transcription Factors

### 4.1. MYC2 as a Central Regulatory Hub Gene Across and Within Species

*MYC2* transcription factors are known to regulate the expression of more than 1300 genes and play a pivotal role as core regulators in the JA signaling pathway [109]. Their broad involvement in various biological processes, including the regulation of secondary metabolism, hormonal crosstalk, and plant responses to abiotic and biotic stresses, has led to the widely accepted view that *MYC2* functions as a central transcriptional hub. However, this inference is often drawn from scattered observations across multiple plant species. To determine whether *MYC2* indeed acts as a central regulator, assessing its roles within individual species is important.

Evidence from several models and crop plants supports the multifaceted regulatory role of *MYC2* within individual species. Beyond its well-established functions in plant growth and development [19], *AtMYC2* in *A. thaliana* regulates flavonoid biosynthesis, drought and salt tolerance, ABA signaling, and SA-JA antagonism by interacting with a range of downstream targets, including *ERD1*, *ABA2*, *HMA2*, *GL3*, and *NPR1* [73,100,114,134]. Similarly, in *tomato*, *SlMYC2* controls steroidal glycoalkaloid biosynthesis, cold and drought resistance, melatonin and polyamine metabolism, and volatile production activating genes such as *SlGSTU24*, *SlBAM3*, *SlADC1*, *SlERF.B8*, and *SlLOX5a* [30,92,93,99]. These cases illustrate that *MYC2* acts as an integrative regulatory factor capable of synchronizing multiple environmental signals and developmental programs within a single plant species.

Comparative studies across plant species show that *MYC2* transcription factors are highly conserved in their domain and JA-responsiveness, particularly in their ability to bind G-box/E-box *cis*-elements. However, their downstream regulatory targets are often species-specific. For instance, *MYC2* regulates nicotine biosynthesis in *N. tabacum* [23], artemisinin in *A. annua* [45], taxol in *Taxus* [56], ginkgolides in *G. biloba* [65], and pyrethrins in *T. cinerariifolium* [34]. These compounds are biosynthesized in a species-dependent manner, indicating that *MYC2* has been independently recruited to regulate diverse metabolic pathways across species.

Together, these findings support a model in which *MYC2* transcription factors serve as evolutionarily conserved regulatory switches that control distinct specialized metabolic pathways in different plant species. This regulatory versatility makes *MYC2* an attractive target for metabolic engineering. However, such broad regulatory roles also raise important concerns about pleiotropic effects when manipulating *MYC2* function.

### 4.2. Balancing Growth and Stress Resistance: The Dilemma of MYC Transcription Factors in Plants

Although *MYC2* plays a central role in coordinating JA-mediated defense and specialized metabolism, its activation is frequently associated with growth inhibition. This growth–defense trade-off poses a significant challenge for its direct application in metabolic engineering and the development of stress-resilient cultivars.

One of the underlying causes of this dilemma is the dual regulatory nature of *MYC2*. It functions as a molecular switch that activates secondary metabolite biosynthetic genes and induces transcriptional repressors involved in metabolic downregulation, thereby maintaining cellular homeostasis [142]. Upon accumulation of JA-Ile, JAZ proteins are ubiquitinated and degraded, releasing *MYC2* to activate defense-related genes. Concurrently, JA signaling stabilizes BPM proteins, which serve as adaptors for Cullin3-based E3 ubiquitin ligases and promote the proteasomal degradation of *MYC2* itself. This negative feedback mechanism ensures a tightly controlled transcriptional output and prevents overactivation of the JA response [16,143].

Beyond molecular feedback, *MYC2* exhibits pronounced pleiotropy, impacting both defense responses and plant development. In rice, *MYC2* upregulates GA catabolism genes, such as *GA2ox3* and *GA2ox7*. This suppresses GA levels, prioritizing resources for defense at the expense of growth [139]. Similarly, JA treatments inhibit GA biosynthesis and activate GA catabolism via *miR5998* and *MYC2*, further reducing endogenous GA levels to curtail growth [144]. In *A. thaliana*, *jaz*-deficient mutants that exhibit constitutively active *MYC2* signaling display stunted growth, including reduced shoot and root size, smaller fruits, and lower seed yield. *MYC2*, together with *MYC3* and *MYC4*, negatively regulates seed size, weight, and storage compound accumulation, while triple mutants produce larger, heavier seeds [145,146]. Furthermore, JA-related components like COI1, MED25, and MYC2 restrict seed size, with JA treatment reducing seed growth in a COI1-dependent manner [147]. MYC3/4 also restricts seed growth by impeding seed coat cell proliferation and elongation, collaborating with the KIX-PPD complex to regulate seed size [148].

These findings emphasize that direct overexpression or knockout of *MYC2* may result in undesirable developmental trade-offs, such as reduced biomass or fertility. Therefore, biotechnological applications must move beyond simplistic manipulations of *MYC2* expression and adopt more refined strategies that preserve stress resistance while minimizing growth penalties.

One promising direction is the use of tissue-specific or inducible promoters to restrict *MYC2* expression to particular organs or stress conditions, thereby limiting its activity to scenarios where defense activation is most beneficial. In parallel, synthetic promoter engineering can be used to design *MYC2*-responsive regulatory elements that drive the expression of only selected downstream genes, enabling targeted activation of secondary metabolic pathways without interfering with core developmental processes. Moreover, recent advances in CRISPR/Cas9-based transcriptional control systems allow for precise activation or repression of individual *MYC2* target genes, offering another layer of regulatory specificity. In addition, protein engineering approaches could be employed to modulate *MYC2* interactions with co-regulators such as *MED25*, or to modify its degradation motifs, thereby altering its stability and transcriptional specificity. Finally, manipulation of *MYC2*-interacting repressors, such as specific JAZ or TPL family members, may allow for fine-tuned redirection of *MYC2* activity toward beneficial regulatory branches, while minimizing unwanted pleiotropic effects.

In conclusion, while *MYC2* represents a powerful regulatory node for enhancing secondary metabolism and stress tolerance, its complex feedback mechanisms and broad physiological roles necessitate precision engineering. A deeper understanding of *MYC2* spatiotemporal activity and interaction networks will be essential to fully exploit its potential in developing resilient and productive crops.

## 5. Conclusions

Extensive research on *MYC2* has significantly advanced our understanding of plant responses to environmental stresses and the regulation of secondary metabolism. *MYC2* functions as a central regulatory hub, integrating multiple signaling pathways—especially those mediated by JA to modulate gene expression associated with plant adaptation. This review highlights the pivotal roles of *MYC2* in promoting secondary metabolite biosynthesis and enhancing stress resilience, traits that are essential for sustainable agriculture. However, efforts to manipulate MYC2-mediated pathways face several challenges, including trade-offs between growth and defense, as well as the risk of inducing potentially deleterious gene expression. Addressing these limitations will require precise, spatiotemporal regulation rather than constitutive overexpression. Future research should focus on identifying *MYC2* interaction partners within specific genetic and epigenetic networks, as well as elucidating its regulation of key metabolic pathways. The integration of genome editing technologies such as CRISPR-Cas9 with multi-omics approaches offers promising avenues for fine-tuning *MYC2* function across diverse plant species. Leveraging advanced genetic, molecular, and biotechnological tools to optimize *MYC2* activity could facilitate a more effective balance between plant growth and defense responses, ultimately contributing to enhanced crop productivity and resilience.

## Data Availability

Data sharing is not applicable.
